# Uric acid is an independent risk factor for carotid atherosclerosis in a Japanese elderly population without metabolic syndrome

**DOI:** 10.1186/1475-2840-11-2

**Published:** 2012-01-10

**Authors:** Shuzo Takayama, Ryuichi Kawamoto, Tomo Kusunoki, Masanori Abe, Morikazu Onji

**Affiliations:** 1 Department of Community Medicine, Ehime University Graduate School of Medicine; Ehime 791-0295, Japan; 2 Gastroenterology and Metabology, Ehime University Graduate School of Medicine; Ehime 791-0295, Japan; 3 Department of Internal Medicine, Seiyo Municipal Nomura Hospital, Ehime 797-1212, Japan

**Keywords:** uric acid, metabolic syndrome, carotid atherosclerosis, cardiovascular risk factor, gender

## Abstract

**Background:**

Carotid intima-media thickness (IMT) is an useful surrogate marker of cardiovascular disease. Associations between uric acid (UA), metabolic syndrome (MetS) and carotid IMT have been reported, but findings regarding the relationship have been inconsistent.

**Methods:**

A total of 1,579 Japanese elderly subjects aged ≥65 years {663 men aged, 78 ± 8 (mean ± standard deviation) years and 916 women aged 79 ± 8 years} were divided into 4 groups according to UA quartiles. We first investigated the association between UA concentrations and confounding factors including MetS; then, we assessed whether there is an independent association of UA with carotid IMT and atherosclerosis in participants subdivided according to gender and MetS status.

**Results:**

Carotid IMT was significantly increased according to the quartiles of UA in both genders without MetS and women with MetS. Multivariate logistic regression analysis showed that odds ratio (OR) {95% confidence interval (CI)} in men for carotid atherosclerosis was significantly increased in the third (OR, 1.75; 95% CI, 1.02-3.02), and fourth quartiles (OR, 2.01; 95% CI, 1.12-3.60) of UA compared with that in the first quartile of UA, and the OR in women was significantly increased in the fourth quartile (OR, 2.10; 95% CI, 1.30-3.39). Similarly, the ORs were significantly associated with increasing quartiles of UA in both genders without MetS, but not necessarily increased in those with MetS.

**Conclusions:**

UA was found to be an independent risk factor for incidence of carotid atherosclerosis in both genders without MetS.

## Introduction

Metabolic syndrome (MetS) is a cluster of metabolic abnormalities defined as the clustering of several cardiovascular risk factors in an individual that include visceral obesity, insulin resistance, raised blood pressure (BP), hypertriglyceridemia, low high-density lipoprotein (HDL) cholesterolemia, and impaired fasting plasma glucose (FPG) [1,2], and that it is a predictor of cardiovascular disease (CVD) [3-5]. Population-based studies have shown that MetS is quite common, affecting 13.3~24.4% of Japanese men ≥30 years of age [6], and its prevalence is increasing with the continuous increase in obesity prevalence in Japan.

Uric Acid (UA) is the metabolic end product of purine metabolism in humans; excess accumulation can lead to various diseases [7]. Many previous studies have shown that increased UA levels are also associated with components of MetS and often accompanied by obesity, raised BP [8], hyperlipidemia [9], glucose intolerance [10], and CVD clustering [11], all of which play a causal role in the pathogenesis of CVD. Thus, UA seems to be merely an independent risk factor or marker for atherosclerosis [12,13]. However, its importance as a risk factor is still controversial. Sakata et al. showed that UA levels are not related to increased risk of death from all causes, including CVD and stroke in 8,172 Japanese participants aged ≥30 years [14]. We have demonstrated that UA is more strongly associated with MetS in women than in men, and associated with the prevalence of carotid atherosclerosis {intima-media thickness (IMT) ≥1.0 mm} only in men without MetS but not in men with MetS or in women with or without MetS [15]. Associations between UA, MetS and carotid IMT have been reported, but few of the studies have been conducted in Japanese subjects.

In the present study, we first investigated the association between UA levels and confounding factors including MetS; currently defined as at least 3 of the 5 following conditions: visceral obesity, raised BP, hypertriglyceridemia, low HDL cholesterolemia, and impaired FPG. In addition, we also assessed whether there is an independent association of UA with carotid atherosclerosis in individuals subdivided according to gender and MetS status.

## Materials and methods

### Subjects

Subjects for this investigation were recruited from among consecutive elderly patients aged ≥65 years visited the medical department of Seiyo Municipal Nomura Hospital. Participants with severe cardio-renal or nutritional disorders that would affect BP, lipid and glucose metabolism were excluded. Thus, 1,579 patients were enrolled in the study. All procedures were approved by the Ethics Committee of Seiyo Municipal Nomura Hospital, and written informed consent was obtained from each subject.

### Evaluation of Risk Factors

Information on demographic characteristics and risk factors were collected using the clinical files in all cases. Body mass index (BMI) was calculated by dividing weight (in kilograms) by the square of the height (in meters). We measured BP in the right upper arm of patients in a sedentary posture using a standard sphygmomanometer or an automatic oscillometric BP recorder. Smoking status was defined as the number of cigarette packs per day multiplied by the number of years smoked (pack·year) irrespective of the differentiation of current and past smoking status: never, light (< 20 pack·year), heavy (≥20 pack·year). Alcohol consumption was classified into non-drinker and drinker (available data, n = 1,145). Histories of antihypertensive, antilipidemic, and antidiabetic medication use were also evaluated. Moreover, ischemic stroke and ischemic heart disease were defined as CVD. Total cholesterol (T-C), triglyceride (TG), HDL cholesterol (HDL-C), FPG, and UA were measured under a fasting condition. Low-density lipoprotein cholesterol (LDL-C) level was calculated by the Friedewald formula [16], and patients with TG levels ≥400 mg/dl were excluded. The mean UA was significantly lower in women [5.1 ± 2.0 {mean ± standard deviation (SD)} mg/dl] than in men (5.7 ± 2.0 mg/dl; p < 0.001). Therefore, sex-specific quartiles of UA were used. The mean (range) UA values of 3.5 (0.8 to 4.3), 5.0 (4.4 to 5.5), 6.1 (5.6 to 6.8), and 8.4 (6.9 to 16.1) mg/dl were used for men, and 3.1 (0.5 to 3.8), 4.3 (3.9 to 4.7), 5.3 (4.8 to 6.0), and 7.7 (6.1 to 15.4) were used for women (Table 1).

**Table 1:** Characteristics of subjects

		Men	Quartile of serum uric acid				Women	Quartile of serum uric acid			
				
Characteristics	Uric acid range (mg/dL)	UA-1 0.8-4.3 N = 169	UA-2 4.4-5.5 N = 171	UA-3 5.6-6.8 N = 163	UA-4 6.9-16.1 N = 160	*P *for trend*	UA-1 0.5-3.8 N = 247	UA-2 3.9-4.7 N = 213	UA-3 4.8-6.0 N = 235	UA-4 6.1-15.4 N = 221	*P *for trend*
Age (years)		76 ± 8	78 ± 7	78 ± 8	79 ± 7	0.004	79 ± 8	78 ± 8	79 ± 8	82 ± 8	< 0.001
Body mass index†(kg/m^2^)		21.3 ± 4.6	21.9 ± 3.2	21.9 ± 3.9	21.8 ± 4.0	0.372	21.2 ± 3.6	22.1 ± 3.4	22.8 ± 4.8	22.3 ± 4.8	< 0.001
Smoking status‡(never/light/heavy, %)		26.0/20.7/53.3	32.2/19.9/48.0	36.2/17.8/46.0	35.6/16.9/47.5	0.517	99.2/0.4/0.4	96.2/0.5/3.3	65.7/1.3/3.0	93.7/2.7/3.6	0.043
Alcohol consumption¶, N (%)		78 (67.8)	70 (60.9)	65 (59.1)	65 (66.3)	0.470	1 (0.6)	3 (1.8)	6 (3.3)	5 (2.8)	0.286
Systolic blood pressure (mmHg)		132 ± 22	141 ± 22	139 ± 24	136 ± 25	0.003	138 ± 21	139 ± 20	138 ± 21	136 ± 28	0.552
Diastolic blood pressure (mmHg)		76 ± 14	78 ± 13	77 ± 13	76 ± 14	0.672	76 ± 13	76 ± 11	76 ± 13	72 ± 16	0.001
Antihypertensive medication, N (%)		65 (38.5)	75 (43.9)	88 (54.0)	82 (51.3)	0.019	116 (47.0)	105 (49.3)	140 (59.6)	146 (66.1)	< 0.001
Total cholesterol (mg/dl)		167 ± 46	168 ± 42	164 ± 41	171 ± 45	0.505	190 ± 40	188 ± 42	193 ± 43	181 ± 44	0.025
LDL-cholesterol (mg/dl)		102 ± 38	104 ± 33	95 ± 35	105 ± 38	0.061	118 ± 33	114 ± 35	120 ± 37	110 ± 35	0.016
Triglyceride (mg/dl)		68 (52-93)	73 (56-98)	76 (57-112)	89 (69-127)	< 0.001	69 (54-93)	80 (59-109)	92 (69-124)	94 (67-120)	< 0.001
HDL-cholesterol (mg/dl)		49 ± 19	47 ± 15	51 ± 18	45 ± 15	0.024	57 ± 17	56 ± 18	53 ± 15	51 ± 17	< 0.001
Antilipidemic drug use, N (%)		9 (5.3)	7 (4.1)	8 (4.9)	8 (5.0)	0.959	17 (6.9)	13 (6.1)	17 (7.2)	26 (11.8)	0.115
Fasting plasma glucose (mg/dl)		119 (98-152)	112 (95-141)	113 (96-150)	115 (96-139)	0.404	112 (97-146)	108 (94-140)	109 (92-130)	118 (97-155)	0.061
Antidiabetic medication, N (%)		50 (29.6)	35 (20.5)	29 (17.8)	37 (23.1)	0.063	47 (19.0)	48 (22.5)	43 (18.3)	59 (26.7)	0.112
Metabolic syndrome, N(%)		32 (18.9)	50 (29.2)	44 (27.0)	54 (33.8)	0.022	62 (25.1)	71 (33.3)	87 (37.0)	97 (43.9)	< 0.001
Cardiovascular disease, N (%)		65 (38.5)	85 (49.7)	82 (50.3)	83 (51.9)	0.055	87 (35.2)	84 (39.4)	97 (41.3)	91 (41.2)	0.448
Ischemic stroke, N (%)		59 (34.9)	74 (43.3)	73 (44.8)	75 (46.9)	0.131	77 (31.2)	73 (34.3)	78 (33.2)	69 (31.2)	0.869
Ischemic heart disease, N (%)		11 (6.5)	15 (8.8)	17 (10.4)	16 (10.0)	0.592	15 (6.1)	16 (7.5)	21(8.9)	30 (13.6)	0.031

LDL, low-density lipoprotein; HDL, high-density lipoprotein. Plus-minus values are means ± standard deviation. †Body mass index was calculated using weight in kilograms divided by the square of the height in meters. ‡Smoking status: daily consumption (pack)×duration of smoking (year): never, light (< 20 pack year), and heavy (≥20 pack·year). ¶Alcohol consumption was classified into non-drinker and drinker (available data, n = 1,145). Data for triglycerides and fasting plasma glucose were skewed, and are presented as the median (interquartile range) and were log-transformed for analysis. *P-value: ANOVA or χ^2^-test.

### Ultrasound image analysis

An ultrasonograph (Hitachi EUB-565, Aloka SSD-2000, or Prosound-α6) equipped with a 7.5 MHz linear type B-mode probe was used by a specialist in ultrasonography to evaluate sclerotic lesions of the common carotid arteries on a day close to the day of blood biochemistry analysis (within 2 days). Patients were asked to assume a supine position, and the bilateral carotid arteries were observed obliquely from the anterior and posterior directions. We measured the thickness of the intima-media complex (IMT) on the far wall of the bilateral common carotid artery about 10 mm proximal to the bifurcation of the carotid artery (as the image at that site is more clearly depicted than that at the near wall) [17,18] as well as the wall thickness near the 10 mm point on a B-mode monitor. We then used the mean value for analysis. Carotid plaques were considered as localized thickening and the echo luminance included those equal to high echogenic structures encroaching into the vessel lumen through common carotid artery to carotid bifurcation [17]. Carotid atherosclerosis was defined as IMT ≥1.0 mm or plaque lesion [17-19].

### MetS

We applied condition-specific cutoff points for MetS based on the modified criteria of the National Cholesterol Education Program's Adult Treatment Panel (NCEP-ATP) III report [2]. MetS was defined as subjects with at least three or more of the following five conditions: 1) obesity: BMI ≥25.0 kg/m^2 ^according to the guidelines of the Japanese Society for the Study of Obesity (waist circumference was not available in this study) [20,21]; 2) raised BP with a systolic BP (SBP) ≥130 mmHg and/or diastolic BP (DBP) ≥85 mmHg, and/or current treatment for hypertension; 3) hypertriglyceridemia with a TG level ≥150 mg/dL; 4) low HDL cholesterolemia with a HDL-C level < 40 mg/dL in men and < 50 mg/dL in women and/or current treatment for dyslipidemia; and 5) impaired fasting glucose with a FPG level ≥100 mg/dL and/or current treatment for diabetes.

### Statistical analysis

All values are expressed as the mean ± standard deviation (SD), unless otherwise specified, and in the cases of parameters with non-normal distributions (TG, FPG), the data are shown as median (interquartile range) values. In all analyses, parameters with non-normal distributions were used after log-transformation. Statistical analysis was performed using PASW Statistics 17.0 (Statistical Package for Social Science Japan, Inc., Tokyo, Japan). The differences of means and prevalence among the groups were analyzed by ANOVA and χ^2 ^test, respectively, and A *post hoc *analysis was performed with Dunnett's test. A multivariate logistic regression analysis was employed to evaluate the contribution of confounding risk factors (e.g., age, BMI, smoking status, SBP, DBP, antihypertensive medication, LDL-C, HDL-C, TG, antilipidemic medication, FPG, antidiabetic medication, and history of CVD) for carotid atherosclerosis. A value of p < 0.05 was considered significant.

## Results

### Background of Subjects

Table 1 shows the clinical characteristics of the 663 male and 916 female subjects. Ages of the enrolled subjects ranged with a mean of 79 ± 8 years (men, 78 ± 8 years; women, 79 ± 8 years; *P *< 0.001). In men, age, prevalence of antihypertensive medication, and TG showed a gradual increasing trend. In women, age, prevalence of antihypertensive medication, TG and prevalence of Ischemic heart disease showed a gradual increasing trend and HDL-C showed a gradual decrease. The prevalence of MetS also showed a gradual increasing according to the UA quartiles in both genders. There were no inter-group differences in prevalence of alcohol consumption, FPG, prevalence of antidiabetic and antilipidemic medication in both genders.

### Carotid atherosclerosis of subjects according to quartile of UA

**Figure 1:**
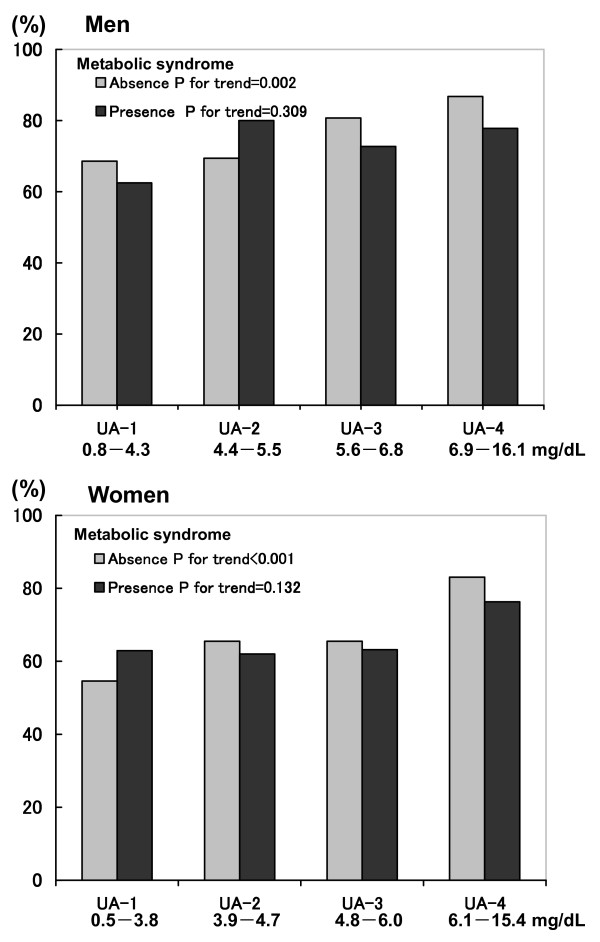
**Prevalence of carotid atherosclerosis according to quartile of uric acid (UA) and metabolic syndrome by gender**. The prevalence of carotid atherosclerosis was also significantly increased with increased UA quartile only in both genders without metabolic syndrome.

As shown in Table 2, carotid IMT was significantly increased according to the UA quartiles in both genders without MetS and in women with MetS, but was not necessarily increased in men with MetS. As shown in Figure 1, the prevalence of carotid atherosclerosis was significantly increased with increased UA quartile in both genders without MetS.

**Table 2:** Carotid intima-media thickness of subjects according to quartile of uric acid and metabolic syndrome by gender

	Quartile of serum uric acid				
		
Uric acid range Men (mg/dL) Women(mg/dL)	UA-1 0.8-4.3 0.5-3.8	UA-2 4.4-5.5 3.9-4.7	UA-3 5.6-6.8 4.8-6.0	UA-4 6.9-16.1 6.1-15.4	***P***for trend*
All subjects					
Men, N = 663	1.02 ± 0.20 #	1.06 ± 0.26	1.03 ± 0.20 §	1.13 ± 0.27	< 0.001
Women, N = 916	0.97 ± 0.18 #	0.98 ± 0.20 #	0.99 ± 0.19 §	1.06 ± 0.25	< 0.001
Subjects without metabolic syndrome (< 3 components of metabolic syndrome)					
Men, N = 483	1.01 ± 0.20 #	1.07 ± 0.29	1.03 ± 0.20 §	1.15 ± 0.28	< 0.001
Women, N = 599	0.95 ± 0.18 §	0.97 ± 0.19 †	0.99 ± 0.20	1.04 ± 0.21	0.002
Subjects with metabolic syndrome (≥3 components of metabolic syndrome)					
Men, N = 180	1.07 ± 0.23	1.04 ± 0.14	1.05 ± 0.19	1.10 ± 0.24	0.487
Women, N = 317	1.01 ± 0.18	0.99 ± 0.22 †	1.00 ± 0.19 †	1.10 ± 0.23	0.004

† *P *< 0.05; § *P *< 0.005; # P < 0.001 vs. the fourth quartile (UA-4). *P-value: ANOVA and Dunnett's test was used for the *pos hoc *analysis.

### Adjusted-odds ratio for carotid atherosclerosis according to quartile of UA and MetS by gender

In Table 3, to examine possible associations between quartiles of UA and the incidence of carotid atherosclerosis after subdivision of the subjects according to MetS status, multivariate logistic regression analysis was performed adjusted for age, gender, BMI, smoking status, SBP, DBP, antihypertensive medication, LDL-C, HDL-C, TG, antilipidemic medication, FPG, antidiabetic medication, and history of CVD. In men, ORs (95% CI) were significantly increased in the third {1.75 (1.02-3.02)} and fourth quartiles {2.01 (1.12-3.60)} of UA compared with that in the first quartile, and in women the OR was significantly increased only in the fourth quartile {2.10 (1.30-3.39)}. Similarly, the ORs were significantly associated with increasing quartiles of UA in both genders without MetS, but not necessarily increased in those with MetS.

**Table 3:** Adjusted-odds ratio (95% CI) for carotid atherosclerosis according to quartile of uric acid and metabolic syndrome by gender

	Quartile of serum uric acid			
	
Uric acid range Men (mg/dL) Women (mg/dL)	UA-1 0.8-4.3 0.5-3.8	UA-2 4.4-5.5 3.9-4.7	UA-3 5.6-6.8 4.8-6.0	UA-4 6.9-16.1 6.1-15.4
All subjects				
Men, N = 663	1 (reference)	1.06 (0.63-1.76)	1.75 (1.02-3.02) †	2.01 (1.12-3.60) †
Women, N = 916	1 (reference)	1.44 (0.94-2.21)	1.29 (0.84-1.98)	2.10 (1.30-3.39)§
Subjects without metabolic syndrome (< 3 components of metabolic syndrome)				
Men, N = 483	1 (reference)	0.92 (0.51-1.67)	2.00 (1.03-3.87) †	2.54 (1.21-5.34) ‡
Women, N = 599	1 (reference)	1.78 (1.06-3.00)†	1.41 (0.83-2.40)	2.54 (1.35-4.78)§
Subjects with metabolic syndrome (≥3 components of metabolic syndrome)				
Men, N = 180	1 (reference)	2.14 (0.67-6.81)	1.55 (0.50-4.83)	2.26 (0.72-7.10)
Women, N = 317	1 (reference)	0.81 (0.36-1.79)	1.12 (0.51-2.49)	1.43 (0.62-3.27)

CI, confidential interval. Adjusted for age, body mass index, smoking status, systolic blood pressure, diastolic blood pressure, antihypertensive medication, LDL-cholesterol, HDL-cholesterol, triglycerides, antilipidemic medication, fasting plasma glucose, antidiabetic medication, and history of cardiovascular disease. † *P *< 0.05; ‡ *P *< 0.01; § *P *< 0.005 vs. the first quartile (UA-1).

### Adjusted-odds ratio of subgroups for carotid atherosclerosis according to quartile of UA by gender

Next, to control potential confounding factors by age, BMI, history of CVD, and medication, the data were further stratified by their values (Table 4). In men, the ORs for carotid atherosclerosis were significant in subgroups of age ≥75 years, BMI ≥ 25 kg/m^2^, no history of CVD, and absence of medication, and in women the ORs were significant in subgroups of age < 75 years, BMI ≥25 kg/m^2^, regardless of history of CVD, presence of medication.

**Table 4:** Adjusted-odds ratio (95% CI) of subgroups for carotid atherosclerosis according to quartile of uric acid by gender

		Men					Women					
	
Uric acid range (mg/dl) Characteristics	N	UA-1 0.8-4.3	UA-2 4.4-5.5	UA-3 5.6-6.8	UA-4 6.9-16.1	*P* for trend*	N	UA-1 0.5-3.8	UA-2 3.9-4.7	UA-3 4.8-6.0	UA-4 6.1-15.4	*P *for trend*
Age												
< 75 years	241	1 (reference)	0.72 (0.33-1.60)	0.99 (0.46-2.14)	1.08 (0.44-2.64)	0.802	293	1 (reference)	1.63 (0.80-3.34)	1.35 (0.65-2.79)	4.11 (1.61-10.5) §	0.020
≥75 years	422	1 (reference)	1.33 (0.66-2.67)	2.76 (1.21-6.29) †	3.00 (1.34-6.70) ‡	0.015	623	1 (reference)	1.49 (0.85-2.63)	1.24 (0.71-2.17)	1.57 (0.87-2.83)	0.400
Body mass index												
<25 kg/m^2^	557	1 (reference)	1.08 (0.62-1.88)	1.79 (0.98-3.26)	1.61 (0.86-2.99)	0.162	729	1 (reference)	1.46 (0.90-2.35)	1.38 (0.85-2.26)	1.51 (0.88-2.59)	0.314
≥25 kg/m^2^	106	1 (reference)	1.54 (0.23-10.3)	3.17 (0.49-20.4)	15.1 (1.60-143) †	0.030	187	1 (reference)	1.92 (0.64-5.78)	1.77 (0.63-4.99)	8.66 (2.44-30.8) #	0.001
History of CVD												
No	348	1 (reference)	1.34 (0.72-2.50)	2.47 (1.26-4.86) ‡	2.02 (1.01-4.06) †	0.037	557	1 (reference)	2.00 (1.18-3.37) †	1.13 (0.68-1.90)	1.95 (1.10-3.45) †	0.016
Yes	315	1 (reference)	0.66 (0.25-1.75)	0.93 (0.33-2.62)	1.98 (0.63-6.28)	0.185	359	1 (reference)	0.87 (0.40-1.85)	1.79 (0.77-4.15)	2.93 (1.08-7.92) †	0.037
Medication												
No	279	1 (reference)	1.23 (0.59-2.54)	2.52 (1.09-5.82) †	3.71 (1.47-9.35) ‡	0.011	333	1 (reference)	1.69 (0.84-3.41)	1.97 (0.93-4.15)	0.95 (0.41-2.18)	0.178
Yes	384	1 (reference)	0.89 (0.41-1.90	1.27 (0.60-2.70)	1.19 (0.54-2.64)	0.786	583	1 (reference)	1.36 (0.77-2.40)	1.15 (0.66-1.99)	2.84 (1.52-5.33)§	0.004

CVD, cardiovascular disease. Medications include antihypertensive, antilipidemic, and antidiabetic medications. Adjusted for age, body mass index, smoking status, systolic blood pressure, diastolic blood pressure, antihypertensive medication, LDL-cholesterol, HDL-cholesterol, triglycerides, antilipidemic medication, fasting plasma glucose, antidiabetic medication, and history of cardiovascular disease. † *P *< 0.05; ‡ *P *< 0.01; § *P *< 0.005; # *P *< 0.001 vs. the first quartile (UA-1).

## Discussion

MetS, representing a cluster of visceral obesity, raised BP, dyslipidemia and glucose intolerance induced by insulin resistance, is a common basis for the development of atherosclerosis, especially CVD [3-5]. In our study, MetS as defined by the modified NCEP-ATP III report criteria was present in 27.1% of men and 34.6% of women. We found 2 results from this cross-sectional data. First, sex-specific UA quartiles are associated with various risk factors and the prevalence of MetS. Second, UA levels might be associated with carotid atherosclerosis independently of other risk factors in both genders without MetS.

Several previous studies have reported possible associations between hyperuricemia and the prevalence of MetS [22-24]. In a prospective study of 8,429 men and 1,260 women aged 20-82 years, 1,120 men and 44 women developed MetS during a mean follow-up of 5.7 years, and men with UA levels ≥6.5 mg/dL had a 1.60-fold increase in risk of MetS (95% CI, 1.34-1.91) as compared with those who had levels < 5.5 mg/dL (P for trend < 0.001), and women with UA levels ≥4.6 mg/dL had a 2.29-fold higher risk of MetS (*P *for trend = 0.02) [22]. These findings suggest that hyperuricemia may be another component of MetS [24]. We thought that sex-specific analyses were also required because at all ages, the UA level is higher in men than in women [22,23]. We cannot explain the underlying mechanism that accounts for the gender difference from this study. A partial explanation for this result could be alcohol consumption, which is more likely to be higher in men, the use of antihypertensive medications such as diuretics, which are known to increase UA levels [25], and the influence of sex hormones [26]. Furthermore, UA tends to positively associate with FPG in subjects without diabetes and negatively associate with FPG in subjects with diabetes [27]. In our study, the analysis was performed after adjusting for FPG, medication, and alcohol consumption (data not shown), but the results were similar. Effects of alcohol consumption or sex hormone require further investigation in the future.

Hyperuricemia is well recognized as a risk factor for atherosclerotic diseases such as CVD [12,13] and carotid atherosclerosis [28-34]. However, whether UA is an independent risk factor for cardiovascular mortality is still a controversy, as previous studies suggest that UA is associated with other cardiovascular risk factors [8-11] despite the strengths of the associations. In cross-sectional screening data from 8,144 individuals, the prevalence of carotid plaque was significantly higher with increasing quartiles of UA level, in men without MetS but not in men with MetS or in women with or without MetS [32]. On the other hand, in a prospective study of 4,966 men (79% white) and 6,522 women (74% white) who were aged 45 to 64 years at baseline, after adjustment for known risk factors that correlate with UA, the association of UA with B-mode ultrasound carotid IMT became non significant in white women and much weaker and not statistically significant in black women and white men [33]. These conflicting findings are partly related to methodological differences and to participant characteristics. In addition, as UA revels relate with increasing numbers of or special metabolic risk factors, the effect of UA on carotid atherosclerosis might become negligible.

The mechanisms by which UA reflects the risk for carotid atherosclerosis are not completely understood even though previous studies have been done in this area, and it is unclear whether high UA levels promote or protect against the development of CVD, or simply act as a passive marker of increased risk. UA regulates critical proinflammatory pathways in vascular smooth muscle cells. Hyperuricemia might be partially responsible for the proinflammatory endocrine imbalance in the adipose tissue and vascular smooth muscle cells [35,36] which is an underlying mechanism of the low-grade inflammation and insulin resistance in subjects with the MetS, and causes dysfunction of endothelial cells. In addition, experimental evidence suggests that adverse effects of UA on the vasculature have been linked to increased chemokine and cytokine expressions, induction of the renin-angiotensin system, and to increased vascular C-reactive protein (CRP) expression [37]. UA also promotes endothelial dysfunction through inactivation of NO and suppression of the proliferation of endothelial cells [38]. Thus, arteriosclerosis induced by hyperuricemia may be a novel mechanism for the development of MetS and carotid atherosclerosis. However, Nieto et al. demonstrated that the higher UA levels seemed associated with elevated total serum antioxidant capacity among individuals with carotid atherosclerosis and be a powerful free radical scavenger in humans [39]. These antioxidant properties of UA could be expected to offer a number of benefits within the cardiovascular system.

We need to be aware of the limitations in interpreting the present results. First, based on its cross-sectional study design, the present result is inherently limited in its ability to elucidate causal relationships between risk factors and carotid atherosclerosis. Second, since all participants were patients, we could not eliminate the possible effects of underlying diseases (e.g., hypertension and diabetes), alcohol consumption, medication (e.g., diuretics, antihyperuricemic and antilipidemic medications) and FPG on the results. Thus, the analysis was performed after adjusting for confounding factors including FPG and medication (e.g., antihypertensive, antilipidemic, and antidiabetic medication). Third, we used BMI ≥25 kg/m^2 ^to classify individuals with visceral obesity because waist circumference measurements were not available, which might have caused an under or over estimation of the effect of visceral obesity on MetS. In fact, the prevalence of MetS in women was higher than those in men and general reports on Japanese [40]. Moreover, secondary prevention interventions after obesity, raised BP, dyslipidemia and diabetes may be successful in reducing risk factors, thus attenuating the observed association of risk factors with diseases. These points need to be addressed again in prospective population-based studies.

In sum, we reported a significant association between the clustering of cardiovascular risk factors known as MetS and UA, and carotid IMT in subjects with risk factors for atherosclerosis. In both genders that did not have MetS, UA was found to be an independent risk factor for the incidence of carotid atherosclerosis. Serum UA confers an increased risk of cardiovascular morbidity, and its identification may thus be important for risk assessment and treatment of patients.

## Competing interests

The authors declare that they have no competing interests.

## Authors' contributions

ST, RK, and MO participated in the design of the study, performed the statistical analysis and drafted the manuscript. TK and MA contributed to the acquisition of data and its interpretation. MO conceived of the study, participated in its design, coordination and helped to draft the manuscript. All authors read and approved the manuscript.
